# Large-scale genomic survey and characterization of mcr genes carried by foodborne *Cronobacter* isolates

**DOI:** 10.1128/msystems.00450-23

**Published:** 2023-09-11

**Authors:** Qianhui Zhu, Jinrui Hu, Na Liu, Heyuan Qi, Xiaoli Du, Zhigang Cui, Yan Sun, Yadong Liu, Songnian Hu, Linhuan Wu, Haijian Zhou, Zilong He, Juncai Ma

**Affiliations:** 1 School of Engineering Medicine, School of Engineering Medicine, Beijing Advanced Innovation Center for Big Data-Based Precision Medicine, Interdisciplinary Innovation Institute of Medicine and Engineering, Beihang University, Beijing, Hebei, China; 2 State Key Laboratory of Microbial Resources, State Key Laboratory of Microbial Resources, Institute of Microbiology, Chinese Academy of Sciences, Beijing, Hebei, China; 3 Institute of Microbiology, University of Chinese Academy of Sciences, Beijing, Hebei, China; 4 State Key Laboratory of Infectious Disease Prevention and Control, State Key Laboratory of Infectious Disease Prevention and Control, National Institute for Communicable Disease Control and Prevention, Chinese Center for Disease Control and Prevention, Beijing, Hebei, China; 5 Microbial Resource and Big Data Center, Microbial Resource and Big Data Center, Institute of Microbiology, Chinese Academy of Sciences, Beijing, Hebei, China; Boston College, Chestnut Hill, Massachusetts, USA

**Keywords:** *Cronobacter*, mcr genes, whole-genome sequencing, foodborne, flanking structures

## Abstract

Antibiotic resistance has become a worldwide public health and biosafety problem. The clinical efficacy of colistin was jeopardized by the report of the first mobile colistin resistance (mcr) gene, and subsequently, the mcr-gene family of mcr-1 to mcr-10 has been identified. *Cronobacter* is an emerging foodborne opportunistic pathogen, which can cause neonatal meningitis, bacteremia, and necrotizing enterocolitis (NEC) by contaminating food. However, the entire picture of foodborne *Cronobacter* carriage of the mcr genes is not known. Here, we investigated the mcr genes of *Cronobacter* isolates (*N* = 877) by whole-genome sequencing. We found 133 of 877 previously undescribed *Cronobacter* isolates carrying mcr genes. Further genomic analysis revealed that these mcr genes mainly belonged to the mcr-9 and mcr-10. Genomic analysis of the flanking structures of mcr genes revealed that two core flanking structures were prevalent in foodborne *Cronobacter* isolates, and the flanking structure carrying IS1R was found for the first time in this study. These findings suggest that foodborne *Cronobacter* isolates carrying the mcr genes already pose a threat to human health.

**IMPORTANCE:**

*Cronobacter* is an emerging foodborne opportunistic pathogen, which can cause neonatal meningitis, bacteremia, and NEC by contaminating food. However, the entire picture of foodborne *Cronobacter* carriage of the mcr genes is not known. Here, we investigated the mcr genes of *Cronobacter* isolates by whole-genome sequencing and found 133 previously undescribed *Cronobacter* isolates carrying mcr genes. Further genomic analysis revealed that these mcr genes mainly belonged to the mcr-9 and mcr-10. Genomic analysis of the flanking structures of mcr genes revealed that two core flanking structures were prevalent in foodborne *Cronobacter* isolates, and the flanking structure carrying IS1R was found for the first time in this study.

## INTRODUCTION

Antibiotic resistance has become a worldwide public health and biosafety problem. Colistin has become increasingly significant as the last line of defense against fatal infections caused by multidrug-resistant (MDR) Gram-negative bacteria (1, 2). Unfortunately, the clinical efficacy of colistin was jeopardized by the report of the first mobile colistin resistance (mcr) gene, mcr-1, in 2015, and subsequently, the mcr-gene family of mcr-1 to mcr-10 has been identified (3 – 12). All of the MCR proteins are phosphoethanolamine (PEA) transferases, which catalyze the attachment of PEA to lipopolysaccharides (LPS)-lipid A, resulting in a decrease in the negative charge of LPS due to structural changes in lipid A and hence resistance to colistin (13).

Following the first mcr-1 gene discovery in China, isolates carrying mcr genes have been reported across worldwide (14, 15). The mcr-1 and mcr-9 were the most widely disseminated and identified in all colistin resistance isolates from 61 and 40 countries across six continents, respectively (14). A wide prevalence and distribution of mcr genes were demonstrated on all continents in various species of Gram-negative bacteria (15). *Cronobacter* is an emerging foodborne opportunistic pathogen, which can cause neonatal meningitis, bacteremia, and necrotizing enterocolitis by contaminating food such as powdered infant formula (PIF) with a very high fatality rate of 10%–40% (16 – 19). The regulatory authorities formulated a series of regulatory policies for PIF. In 2004 and 2006, the World Food and Agriculture Organization and the World Health Organization jointly categorized *Cronobacter* as one of the Category A pathogenic bacteria, that is, there is clearly evidence of causality, and it may be detected in the contaminated PIF. Meanwhile, countries are reducing *Cronobacter* exposure and infection through constant revision of specific microbial criteria for PIF. For example, China’s current standards (GB 25596–2010, GB 10765–2010) have clear regulations on the detection amount of *Cronobacter* in PIF. The mcr genes have only been found on a few occasions in *Cronobacter*; however, the identification of the colistin-resistance genes mcr-1 and mcr-10 in *Cronobacter sakazakii* has highlighted the importance of antibiotic resistance in this species (20, 21). It poses a threat to newborn health and food safety. Further research into the genes encoding antibiotic resistance in *Cronobacter* isolates is a high priority for public health studies.

In this study, we collected foodborne *Cronobacter* isolates carrying the mcr genes from several Chinese provinces and performed whole-genome sequencing. We analyzed the evolutionary relationships, identification of the mcr genes, flanking structures and comparative genomic analysis of these strains with human-derived *Cronobacter* isolates. This study provides a new insight into the colistin-resistance genes of *Cronobacter* and raises concern that plasmid-mediated MDR *Cronobacter* may be a threat to infant health. Enhanced surveillance of antimicrobial drug-resistant *Cronobacter* is warranted.

## MATERIALS AND METHODS

### Sample collection and strains isolation

This study included 133 previously undescribed *Cronobacter* isolates (Table S1). They were isolated from 19 provinces in China between 2006 and 2019 by using the recommended methods in Chinese national standards (22). The sources of these isolates were special dietary food (*n* = 65), milk dairy products (*n* = 44), grain food products (*n* = 14), condiments (*n* = 5), beverages (*n* = 2), cocoa products (*n* = 1), and others (*n* = 2). The Chromogenic *Cronobacter* Isolation Agar (CCI; Oxoid, CM1122, Basingstoke, Hampshire, England) was used to screen *Cronobacter* positive samples. Then one to three typical blue-green single colonies were picked on each plate and repurified by CCI. Primary screening strains were cultured by tryptone soya agar. A real-time quantitative PCR method was used to confirm the *Cronobacter* isolates based on the macromolecular synthesis operon.

### Library construction and whole-genome sequencing

We took the frozen strains out of the −80°C refrigerator and inoculated them into the solid medium. Genomic DNA was extracted from pure cultures using lysozyme, mutanolysin, and RNase A in Tris-EDTA (TE) buffer at 37°C for 60 min, followed by lysis in proteinase K, SDS, and NaCl for 60 min at 55°C. The DNA purity was evaluated using the NanoDrop spectrophotometer, and the DNA concentration was determined using a compact fluorimeter (Qubit 3.0; Thermo Fisher Scientific). The genomic DNA was then diluted to 0.2 ng/mL, and the libraries were prepared using a Nextera XT DNA library preparation kit (Illumina, Inc., Cambridge, UK). A Qubit double-stranded DNA high-sensitivity assay kit (Invitrogen) was used to determine the concentration of the sample libraries, and the libraries were pooled and sequenced using the NovaSeq 6,000 system (Illumina Inc., San Diego, CA, USA).

### Bioinformatics analysis and data processing

FastQC (https://www.bioinformatics.babraham.ac.uk/projects/fastqc/) and MultiQC (23) were used for quality control of the raw data. Low-quality reads, primers, and adapters were trimmed off by using Trimmomatic (24). After data preprocessing, the clean reads were assembled into draft genomes by SPAdes (25) (Table S2). Genome annotation was performed by Prokka (26). Taxonomy classification of all the isolates in this study was determined by both the experimental result and computational software Kraken2 (27). The ST typing was determined by the blast results of ffn sequences for each isolates against MLST template sequences in pubMLST website (https://pubmlst.org/data/) with parameters (evalue：1e-10, identity：100%, and query coverage：100%). The plasmid sequences of each draft genomes were identified by using Platon (28).

### Phylogenetic tree construction

Pan-genome analysis of all the isolates in this study was performed by Roary (29). The “core_gene_alignment.aln” file was used as an input file for the phylogenetic tree construction of all the isolates by using FastTree (30). For mcr genes evolutionary tree, the mcr genes were used for multiple sequence alignment by using MUSCLE (31) and generated trees by FastTree (30). All the trees were visualized by iTol (32).

### Identification and genotype of mcr genes

The mcr genes of each isolates were determined by using blast against CARD database (https://card.mcmaster.ca/) (33) with parameters (evalue：1e-5, identity：80%, query coverage：80%, and subject coverage：80%). The genotype of mcr genes in this study was identified by using multiple sequence alignments and phylogenetic tree combined with the known mcr genes (from mcr-1 to mcr-10) in NCBI database.

### Analysis of the flanking structure of mcr genes

The flanking structure of mcr genes was identified by using online tools ISfinder (https://www-is.biotoul.fr/index.php) (34). The contigs/scaffolds contained mcr genes were used as input for subsequent analysis. The flanking structures from the same ST typing were visualized by EasyFig (35).

### Genomics analysis of *Cronobacter* genomes from a human source

According to the metadata, we selected 920 genomes of *Cronobacter* genus from NCBI database (https://ftp.ncbi.nlm.nih.gov/genomes/genbank/bacteria/), including 820 strains of *C. sakazakii*, 80 strains of *Cronobacter malonaticus*, and 20 strains of *Cronobacter turicensis* (Table S3). The bioinformatics analysis was described as above description.

## RESULTS

### Basic information statistics of *Cronobacter* genomes carrying mcr genes in this study

**Fig 1:**
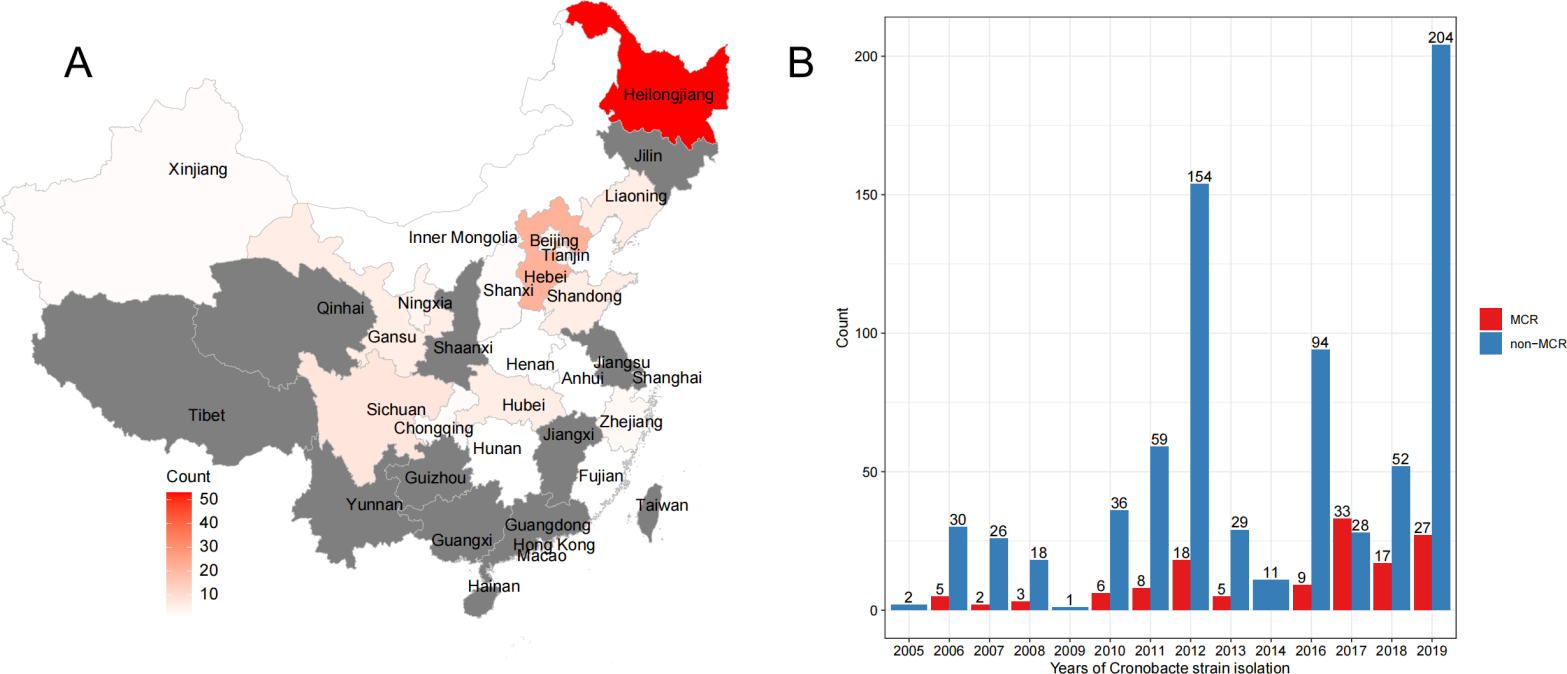
The distribution of *Cronobacter* isolates carrying mcr genes. (A) The geographical distribution of *Cronobacter* isolates carrying mcr genes (gray areas indicate absence). (B) Time-scale distribution of *Cronobacter* isolates carrying mcr genes.

We conducted a genome survey on *Cronobacter* genus isolated from Chinese food samples (total number = 877) by using whole-genome sequencing (Table S4). With the identification of drug-resistance genes (Fig. S1), a total of 99 types of drug-resistance genes were identified, of which 23 had a frequency of more than 95%, mainly from fluoroquinolone antibiotic, aminocoumarin antibiotic, and aminoglycoside antibiotic, as well as from various antibiotic types. In addition, there were 69 types of drug-resistance genes with a frequency of less than 1%; 133 isolates were found to carry the mcr genes, including 121 *C. sakazakii*, 7 *C. malonaticus*, and 5 *C. turicensis*. The 133 strains were isolated from 7 kinds of food samples, including special dietary foods (*n* = 65), milk and dairy products (*n* = 44), grain food products (*n* = 14), condiments (*n* = 5), beverages (*n* = 2), cocoa products (*n* = 1), and others (*n* = 2; Table S4). The 133 strains were collected from 22 regions. On the geographical distribution, most provinces in the north and middle of China have detected *Cronobacter* carrying mcr genes, of which only Heilongjiang Province has detected 51 strains, while some southern provinces, such as Guizhou, Jiangxi, and Guangdong, have not detected such strains (Fig. 1A). The years of sample collection were from 2006 to 2019 (except 2009, 2014, and 2015). On the whole, the number of *Cronobacter* carrying mcr genes detected showed an increasing trend and reached 33 and 27 in 2017 and 2019, respectively, and the detection rate of the mcr gene in *Cronobacter* has also increased over the years (Fig. 1B).

### The genotype of mcr genes in *Cronobacter* genomes

**Fig 2:**
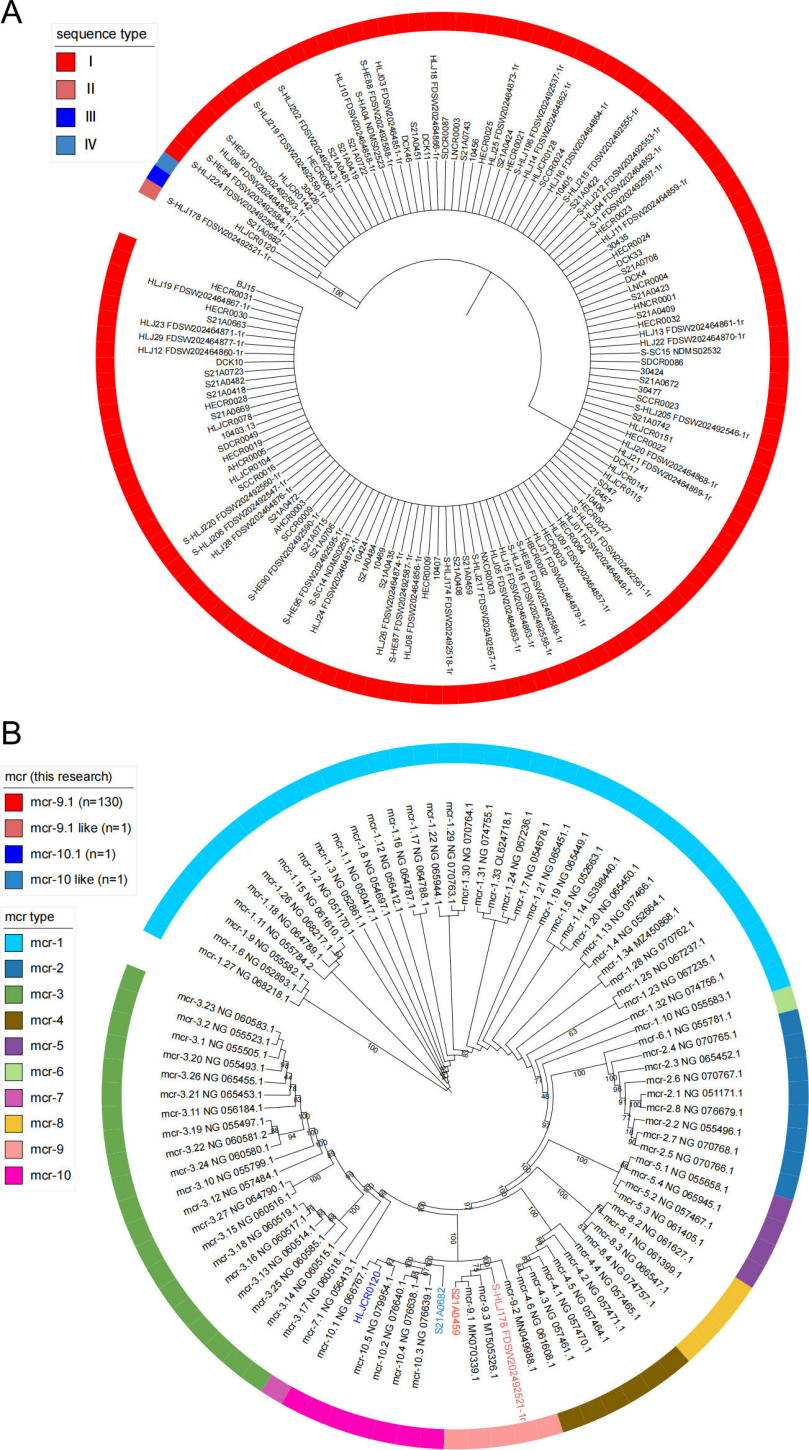
Phylogenetic tree of mcr genes from *Cronobacter* isolates. (A) Phylogenetic tree based on the nucleic acid sequences of 133 mcr genes. (B) The phylogenetic tree based on the four mcr nucleic acid sequences in this study and the known mcr genomic typing sequences.

The phylogenetic tree analysis of the mcr genes in 133 isolates of *Cronobacter* was performed in Fig. 2A; 133 genes were divided into three clades, of which the largest clade contained 130 mcr genes. After multiple sequence alignment analysis, 133 mcr genes displayed four types of nucleic acid variation. In order to further study the genotype of mcr genes, four types of nucleic acid variation (HLJ178_FDSW202492521-1, HLJCR0120, S21A0682, represented by S21A0459 on major clade) and the known mcr sequences (from mcr-1 to mcr-10) downloaded from NCBI database were analyzed by a phylogenetic tree. The results showed that S21A0459 and HLJ178_ FDSW202492521-1 are in the same branch as the subtype of mcr-9, and HLJCR0120 and S21A0682 are in the same branch as the subtype of mcr-10 (Fig. 2B). Multiple sequence alignment showed that the nucleic acid sequence of S21A0459 was identical to mcr-9.1, which meant that 130 mcr genes in the major clade of the phylogenetic tree were all mcr-9.1 (Fig. S2A). On the other hand, HLJ178_ FDSW202492521-1 has 36 bp more sequence at the 5' end of nucleic acid than other mcr-9 (ATGTTTTTACTGGTTACTATTCCGGGAGGTTA), and the rest of FDSW202492521-1 is identical to the mcr-9.1 gene, and the additional sequence at the 5' end has the starting codon “ATG,” but it is not clear how this situation affects the function of the gene. In addition, the multiple sequence alignment results show that the nucleic acid sequences of HLJCR0120 and mcr-10.1 are completely consistent, while the mcr sequence in S21A0682 is in the branch of mcr-10 and the same length as mcr-10.1, but the multiple sequence alignment results show that there are multiple single nucleotide polymorphism (SNP) sites distributed on the whole gene sequence (Fig. S2B), and the similarity between this sequence and mcr-10.1, mcr-10.2, mcr-10.3, mcr-10.4, and mcr-10.5 nucleic acid sequences is 83.7%, 83.6%, 83.6%, 83.7%, and 83.6%, respectively, suggesting that this may be a new type of mcr gene. Finally, 130 mcr-9.1 and 1 mcr-9.1 like, 1 mcr-10.1, and 1 mcr-10 like were identified among 133 isolates of the *Cronobacter*.

### The flanking structure of mcr genes in *Cronobacter* genomes

**Fig 3:**
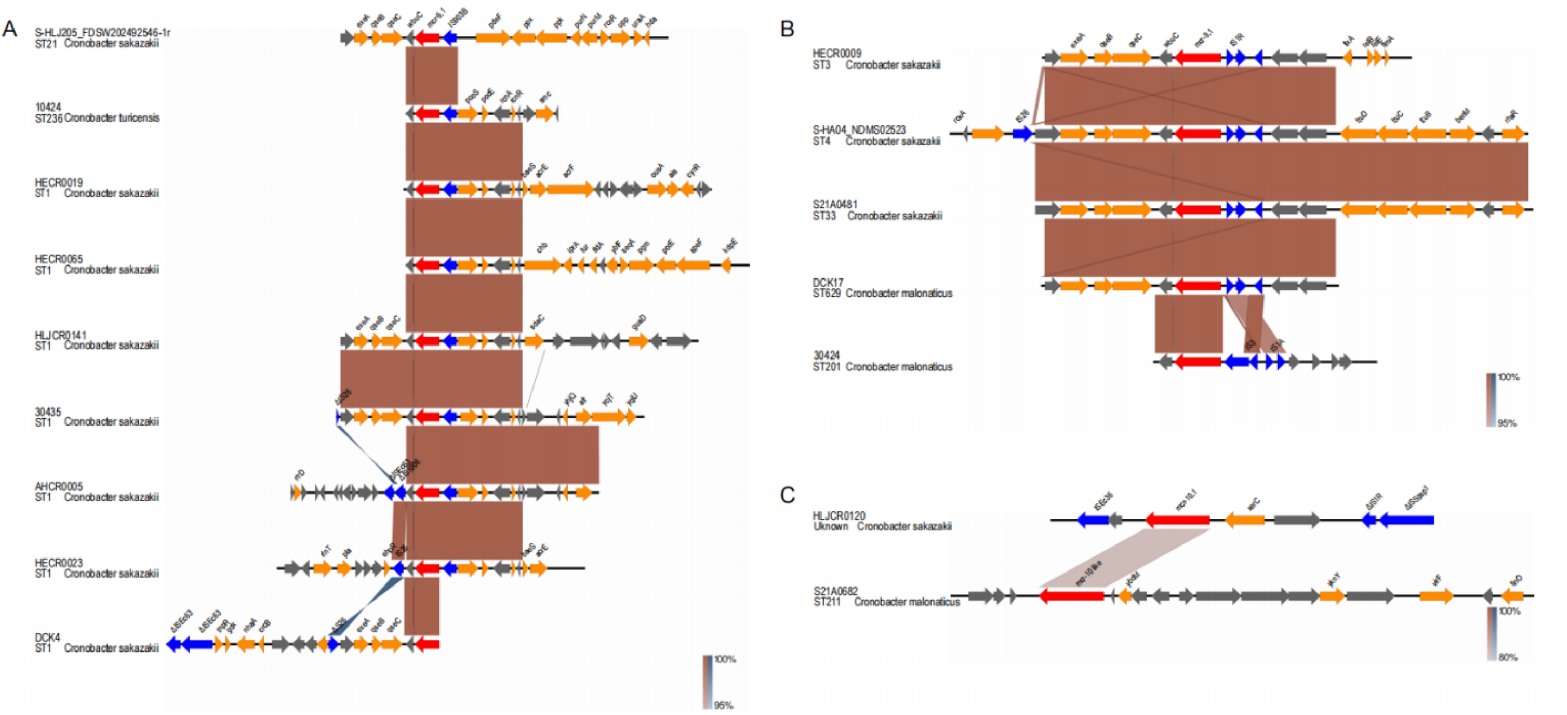
The flanking structures of mcr genes from *Cronobacter* isolates in this study. (A) The flanking structure of the mcr-9.1 genes from *Cronobacter* isolates (the upstream gene is IS903B). (B) The Flanking structure of the mcr-9.1 genes from *Cronobacter* isolates (the upstream gene is IS1R). (C) The flanking structure of the mcr-10 genes from *Cronobacter* isolates.

We analyzed the flanking structure of mcr-9.1 and mcr-10 (due to the influence of genome assembly, the gene structure on both sides of mcr-9.1 like cannot be inferred, and the contig of mcr-9.1 like is only 2,074 bp). After identification of insertion sequence and analysis of flanking structure, it is found that mcr-9.1 mainly has two core flanking structures containing insertion sequence IS903B and IS1R in *Cronobacter*: Hp-exeA-qseB-qseC-wbuC-mcr-9.1-IS903B-pcoS-PcoE-Hp-rcnR and Hp-exeA-qseB-qseC-wbuC-mcr-9.1-IS1R- ΔISEc17-Hp-Hp (Hp represents hypothetical protein). Among the core structures of Hp-exeA-qseB-qseC-wbuC-mcr-9.1-IS903B-pcoS-PcoE-Hp rcnR, the most stable structure is the mcr-9.1-IS903B, i.e., IS903B is located at the upstream of mcr-9.1, and this flank structure is popular in many species of Enterobacteriaceae by using blast against NR database (Fig. 3A). In addition, there is an insertion sequence downstream of mcr-9.1 in some strains. For example, a truncated IS26 is added downstream of mcr-9.1 of strain 30,435, and a truncated IS26 also exists downstream of mcr-9.1 of DCK4; in DCK4 strain, a truncated ISEc603 appeared in the area of about 7 kb of IS26, while the strains AHCR0005 and HECR0023 directly inserted the insertion sequence after the wbuC downstream of mcr-9.1. Two adjacent truncated insertion sequences are inserted downstream of wbuC in AHCR0005 (IS1006 and ISEc63), in which IS1006 and IS26 are insertion sequences of IS6 family, with high homology, the downstream of ISEc63 and the ISEc63 region in DCK4 is highly homologous. HECR0023 strain inserted a complete IS26 after the wbuC downstream of mcr-9.1.

**Fig 4:**
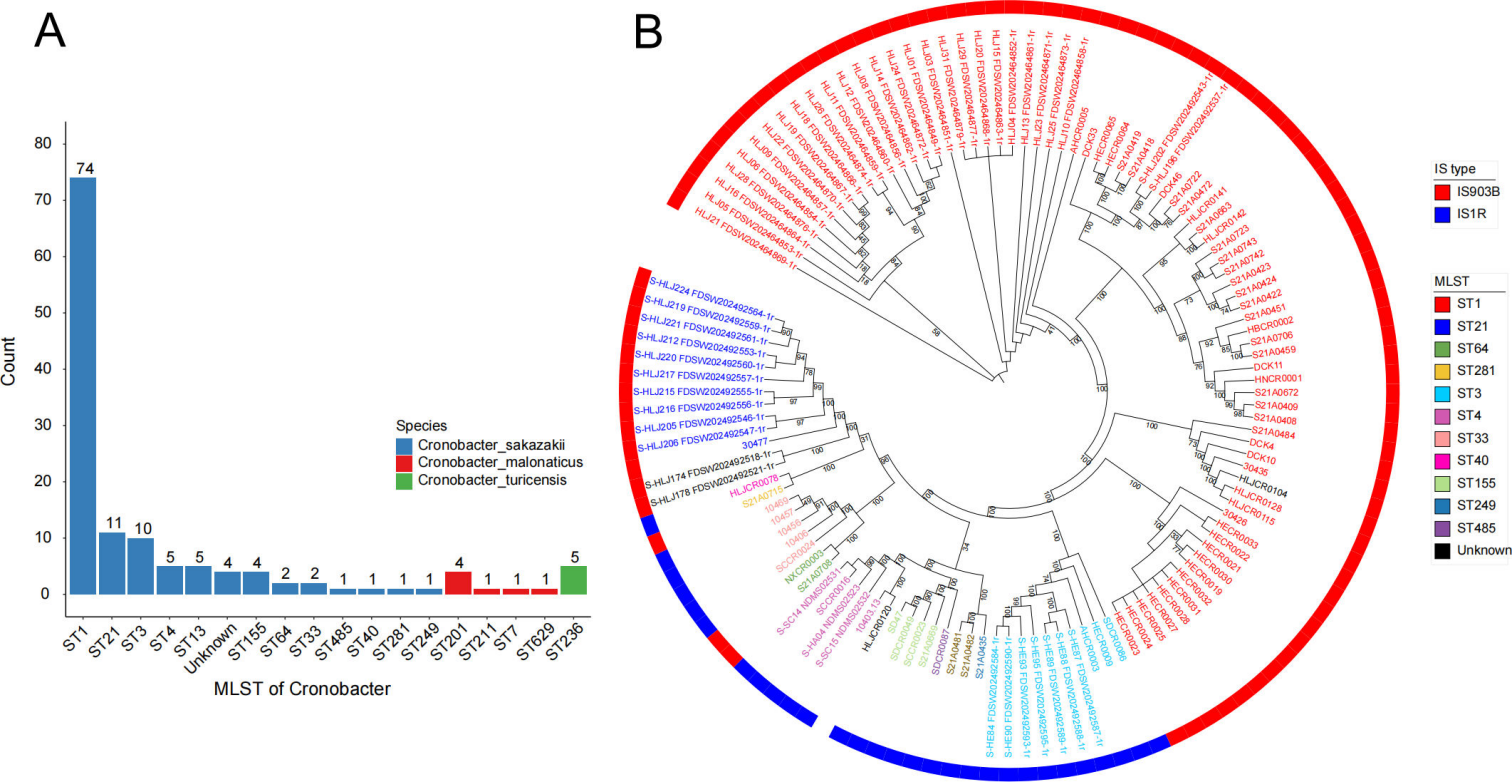
(A) MLST statistics for the genus *Cronobacter* carrying mcr genes in this study. (B) The phylogenetic tree of *Cronobacter sakazakii* isolates.

In the core structure of Hp-exeA-qseB-qseC-wbuC-mcr-9.1-IS1R-ΔISEc17-Hp-Hp, the most stable structure is mcr-9.1-IS1R-ΔISEc17, namely IS1R and truncated ISEc17, is located upstream of mcr-9.1, and this flank structure has not been reported at present (Fig. 3B). Because the genome sequences of some isolates are limited by the genome assembly quality, so it is impossible to analyze the prevalence of its downstream inserted sequence. However, the structure of S-HA04_NDMS02523 is relatively complete. There is an insertion sequence IS26 downstream of mcr-9.1, which is similar to the popular IS26 downstream of mcr-9.1 and its insertion sequence of the same family in the mcr-9.1-IS903B structure. In the strain 30,424 classified into ST201, we found the upstream flanking structure of mcr-9.1, wbuC-mcr-9.1-IS3-IS1A, which is different from the above two popular core flanking structures. The insertion sequence of this structure and the one in mcr-9.1-IS1R-ΔISEc17 is from the same family. The *Cronobacter* genomes in this study have 17 kinds of ST types; 58 of 133 mcr genes are located in chromosome genomes, and the other 75 mcr genes are located in plasmid genomes. According to the statistics of the upstream insertion sequence of mcr-9.1 in *Cronobacter*, almost all the strains of the same ST type have the same insertion sequence (Table 1; Fig. 4A). In the phylogenetic tree of *C. sakazakii*, it was found that the strains carrying the same insertion sequence upstream of mcr-9.1, the strains with similar flanking structure of mcr-9.1, had a closer evolutionary distance. Except that the three strains carrying IS903B were mixed in the strain group of IS1R, and the strains on the same branch of the phylogenetic tree all carried the same insertion sequence (Fig. 4B).

**TABLE 1:** The upstream IS genes of mcr 9.1 genes

Species	MLST	Count	IS type
*Cronobacter sakazakii*	ST1	74	IS*903B*
*C. sakazakii*	ST21	11	IS*903B*
*C. sakazakii*	ST64	2	IS*903B*
*Cronobacter turicensis*	ST236	5	IS*903B*
*C. sakazakii*	ST281	1	IS*903B*
*C. sakazakii*	Unknown	2	IS*903B*
*Cronobacter malonaticus*	ST201	4	IS*1A*/IS*903B*
*C. sakazakii*	ST3	10	IS*1R*
*C. sakazakii*	ST4	5	IS*1R*
*C. malonaticus*	ST7	1	IS*1R*
*C. sakazakii*	ST13	5	IS*1R*
*C. sakazakii*	ST33	2	IS*1R*
*C. sakazakii*	ST40	1	IS*1R*
*C. sakazakii*	ST155	4	IS*1R*
*C. sakazakii*	ST249	1	IS*1R*
*C. sakazakii*	ST485	1	IS*1R*
*C. malonaticus*	ST629	1	IS*1R*

Insertion sequence and flanking structure analysis found that there were two truncated sequences IS1R and ISSgsp1 upstream of mcr-10.1 in HLJCR0120, and an entire insertion sequence ISEc36 downstream, and the insertion sequence was the same as the downstream insertion sequence of mcr-10 of three strains isolated from hospital sewage (Fig. 3C). In addition, the gene upstream of mcr-10.1 in our study and the gene upstream of mcr-10 in the above report are both xerC. Besides this gene, there are no other highly conservative genes upstream and downstream of mcr-10. In the upstream and downstream of mcr-10 like, there is no insertion sequence, and there is no gene homologous to the flanking gene of mcr-10.1.

### Genome survey of *Cronobacter* isolates carrying mcr genes from human source on a public database

We screened 920 whole genomes of *Cronobacter* which were published on the NCBI. A total of seven strains of *C. sakazakii* and six strains of *Cronobacter malonate* carrying the mcr genes were detected (Table 2). Human strains were detected in five countries including Ireland, Canada, the United States, China, and the Czech Republic, of which four strains were detected in China. The strain carrying mcr-10.1 was one of *C. sakazakii* ST8 isolated from human feces in China in 2019, and the other 12 strains carried mcr-9.1. According to the metadata, the strain carrying mcr-9.1 was first isolated in the 1970s, and it was two strains of *C. malonate* ST7 isolated from nasal cavity and feces of clinical patients in the United States. From the flanking structure, the popular mcr-9.1 side flanking structure of human source is similar to that of food source strains. Among them, six strains of *C. sakazakii* ST1, ST21, and ST256 contain mcr-9.1-IS903B in the flanking structure, four of five in *C. malonate* contain mcr-9.1-IS1R in the flanking structure, and the remaining two strains of *C. malonate* ST60 and ST7 failed to analyze the flanking structure because the contig where mcr-9.1 is located is too short.

**TABLE 2:** The basic information of *Cronobacter* carrying mcr genes from human source

Assembly_accession	MLST	Species	mcr type	Host	Country
GCA_002107695.1	ST1	*Cronobacter sakazakii*	*mcr-9.1*	Homo sapiens	Ireland
GCA_008806255.1	ST1	*C. sakazakii*	*mcr-9.1*	Homo sapiens	Ireland
GCA_016576885.1	ST1	*C. sakazakii*	*mcr-9.1*	Homo sapiens	Canada
GCA_016576925.1	ST1	*C. sakazakii*	*mcr-9.1*	Homo sapiens	USA
GCA_013421565.1	ST8	*C. sakazakii*	*mcr-10.1*	Homo sapiens	China
GCA_003207135.1	ST21	*C. sakazakii*	*mcr-9.1*	Homo sapiens	Canada
GCA_003955925.1	ST256	*C. sakazakii*	*mcr-9.1*	Homo sapiens	China
GCA_002093915.1	ST60	*Cronobacter malonaticus*	*mcr-9.1*	Homo sapiens	China: HuBei
GCA_009938865.1	ST7	*C. malonaticus*	*mcr-9.1*	Homo sapiens	China
GCA_016076405.1	ST7	*C. malonaticus*	*mcr-9.1*	Homo sapiens	Czech Republic
GCA_016076495.1	ST7	*C. malonaticus*	*mcr-9.1*	Homo sapiens	Czech Republic
GCA_016076695.1	ST7	*C. malonaticus*	*mcr-9.1*	Homo sapiens	USA: CO
GCA_016077535.1	ST7	*C. malonaticus*	*mcr-9.1*	Homo sapiens	USA: MD

### Phylogenetic analysis of *Cronobacter* genomes from human and food sources

**Fig 5:**
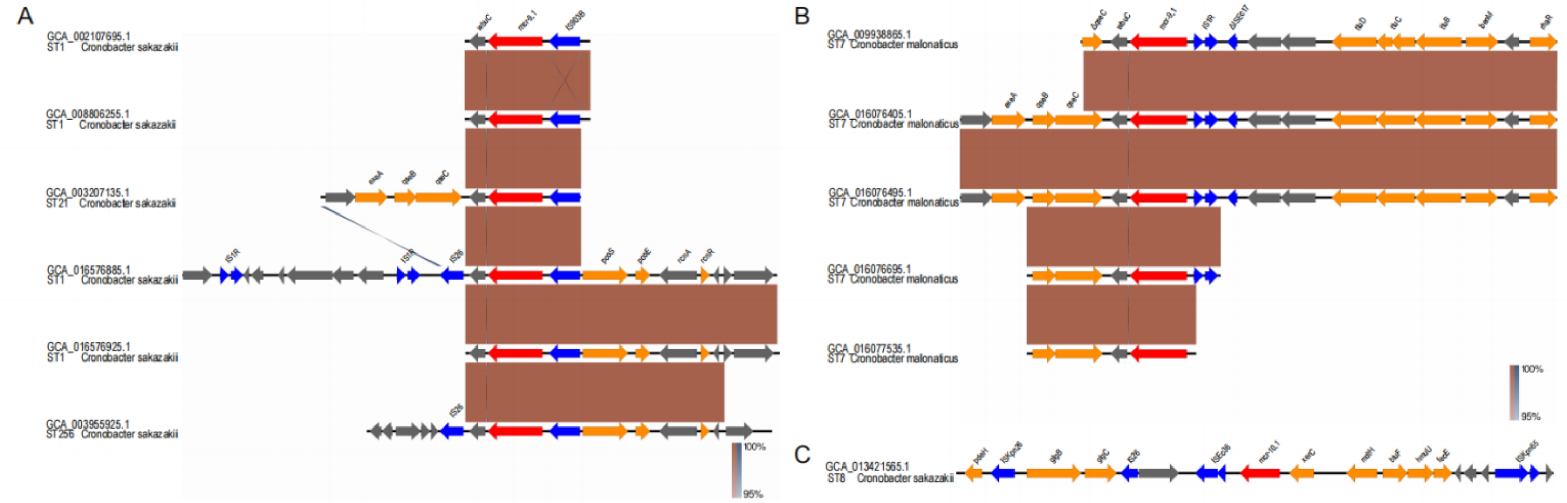
The flanking structures of mcr genes from *Cronobacter* isolates in public human source. (A) The flanking structure of the mcr-9.1 genes from human source (the upstream gene is IS903B). (B) The flanking structure of the mcr-9.1 genes from human source (the upstream gene is IS1R). (C) The flanking structure of the mcr-10.1 genes from human source.

In order to further study the evolutionary relationship of the strains, we conducted phylogenetic tree analysis on 133 strains of food sources and 13 strains of *Cronobacter* from human sources carrying mcr genes (Fig. 5). On the phylogenetic tree, both human and food strains have two types of IS903B and IS1R, and strains from different sources of the same evolutionary tree branch carry the same type of insertion sequence. The number of *C. malonate* is small, and the food and human strains are in the same large branch, but the strains carrying the same insertion sequence evolve closer (in the same small branch). Although the three species of *Cronobacter* are similar in carrying the mcr genes, their genomes are significantly different in the evolutionary relationship. Among them, *C. malonate* and *Cronobacter zurich* are clustered in a large branch. From the source of strains, there is no obvious difference in the evolutionary relationship, which indicates that the evolutionary distance, the carrying mcr genes, and the flanking structures from food source strains and human source strains have obvious similarities.

## DISCUSSION

Pathogens are an important part of food microbial monitoring (36, 37). Once their content exceeds the national standard in food, they may infect consumers and cause infectious diseases (38). The method of food microbial monitoring generally adopts an isolation culture, which has the problems of strict culture conditions and long operation cycle. In the later stage, PCR technology was introduced to conduct rapid detection by adding specific primers of various microorganisms into the reaction system and combining them with the culture method. This method played a certain role in market supervision, but it could not conduct a comprehensive and detailed analysis on the specific pollution source, transmission chain, and drug-resistance gene of bacteria in food. With the development of sequencing technology and the reduction of sequencing cost, the whole genome microbial detection technology based on the next-generation sequencing technology is gradually emerging. The whole genome data contains complete and abundant information of the strain, which can be used to predict the known genes according to the drug-resistance gene database, overcoming the limitations of traditional PCR technology that can only study a small number of genes. At present, China does not use whole-genome sequencing technology to systematically screen large-scale foodborne pathogens. In this study, we use whole-genome sequencing technology to screen 894 strains of foodborne *Cronobacter* isolates collected nationwide. Focusing on the phenotype of polymyxin resistance, 133 strains of *Cronobacter* carrying the mcr resistance genes were screened, and the genotyping, flanking structure, and evolutionary relationship of these strains were studied in detail, filling the gap in this area.

In this study, the detection rate of mcr in *Cronobacter* is as high as 15.48%. However, there is no previous report on the high detection rate of mcr in *Cronobacter*. In recent years, there have been reports of high drug-resistant *Cronobacter* with mcr genes (39 – 41). Relevant reports all believe that *Cronobacter* is a high-risk foodborne pathogen that needs to be focused on in food microbial monitoring (42, 43). The high-frequency detection of mcr genes in foodborne *Cronobacter* in China indicates that we are facing a huge risk to food safety.

In our study, there are 65 strains of *Cronobacter* isolated from special dietary foods, 44 strains from milk and dairy products, 14 strains from food products, and 10 strains from 4 food substrates. This shows that infants and young children face a huge risk of being infected with *Cronobacter* from their main food sources-special dietary foods and milk and dairy products. Due to the weak immune system of newborn infants (PMID:36624474), once they are infected by *Cronobacter* with mcr genes, they are likely to have serious infection complications. According to the time and region of detection of *Cronobacter* carrying mcr genes, this potential hazard began to appear in 2006 in China, and it has spread in 22 provinces in the following decade. This shows that there is a huge risk of polymyxin-resistant bacteria infection in the preparation and processing of special dietary food, milk, and dairy products in China. On the one hand, it may be that the microorganisms carrying mcr genes in raw materials such as cow milk are used as the transmission source to transmit mcr genes to *Cronobacter* contaminated during processing and storage. On the other hand, the use of polymyxin may be involved in the processing and storage of dairy products, which may lead to polymyxin resistance of *Cronobacter*. This suggests that the regulatory authorities should strictly carry out spot checks on microorganisms and drug resistance of upstream raw materials of milk and dairy products and special dietary foods, as well as spot checks on processing plants and products, and strictly control the use of polymyxin in the processing chains at all levels of these two types of foods, so as to reduce the risk of infants and young children being infected with *Cronobacter*, especially those carrying mcr genes.

According to the mcr genes detection of *Cronobacter* from food sources and *Cronobacter* from human sources in the public database in this paper, mcr-9.1 is the prevalent type of *Cronobacter*, and mcr-10.1 is only detected in one strain from each of the two sources. At present, the prevalence of mcr-10.1 in all species is relatively low, and the different flanking structures of mcr-10.1 in this paper are consistent with the description in the literature (44). Through the fine annotation of mcr-9.1, all strains in this paper with IS1R upstream of mcr-9.1 (except those strains that cannot be determined due to the low quality of genome assembly) have a controversial double-grouping regulatory system qseB-qseC. However, there are food strains AHCR0005 and AHCR0023 and human strains GCA_016576885.1 and GCA_003955925.1 all appeared qseB-qseC, and then the insertion sequence directly appeared in the downstream of wbuC. No polymyxin sensitivity test was conducted for the strains in this paper, which could not verify the mediating effect of qseB-qseC, a double-grouping regulatory system, on the expression of mcr-9.1 gene. However, the strains without qseB-qseC indicated that the grouping regulation system qseB-qseC may not be the necessary condition for the existence of mcr-9.1 in the strains. Subsequent studies can focus on such strains to quantitatively analyze the actual role of qseB-qseC in the expression of mcr-9.1 through experiments.

From the perspective of evolutionary relationship, it can be seen that the strains from the two sources carrying the same insertion sequence have a relatively close evolutionary distance, which indicates that the two core flanking structures (Hp-exeA-qseB-qseC-wbuC-mcr-9.1-IS903B-pcoS-pcoE-Hp-rcnR and Hp-exeA-qseB-qseC-wbuC-mcr-9.1-IS1R-ΔISEc17-Hp-Hp) may be used in the transmission of mcr-9.1 in *Cronobacter*, respectively. The main way for *Cronobacter* isolates to infect human is through food transmission. There are two ways for *Cronobacter* to carry the polymyxin resistance gene: one is to overuse polymyxin to treat infection, which leads to drug resistance; the other is horizontal gene transfer by other polymyxin-resistant bacteria. In our study, the high-detection rate of mcr-9.1 in food source strains and the similar distribution of mcr-9.1’s flanking structure in human and food source strains indicate that mcr-9.1 in *Cronobacter* may have entered the human body when the human body is infected by *Cronobacter*. It is speculated that the main reason for the polymyxin resistance of *Cronobacter* in human body is that the foodborne *Cronobacter* carrying mcr-9.1 infects humans through the food chain.

### Conclusion

In summary, we identified 133 foodborne *Cronobacter* isolates carrying the mcr genes by genomic screening (*N* = 877). Further genomic analysis revealed that these mcr genes mainly belonged to the mcr-9 and mcr-10 genes. Analysis of the flanking structures of mcr revealed that two core flanking structures were prevalent in foodborne *Cronobacter*, and the flanking structure carrying IS1R was found for the first time in this study. These foodborne *Cronobacter* are mainly isolated from special diets and dairy products, which pose a serious threat to human health and should be subject to enhanced control of food microorganisms.

## Data Availability

The whole genome sequence data reported in this paper have been deposited and are publicly accessible in the Genome Warehouse in National Genomics Data Center, Beijing Institute of Genomics, Chinese Academy of Sciences/China National Center for Bioinformation (https://ngdc.cncb.ac.cn/gwh), under the accession numbers given in Table S2.

## SUPPLEMENTAL MATERIAL

Figure S1 msystems.00450-23-s0001.pdf Antibiotic resistance profile of *Cronobacter* isolates.

Figure S2 msystems.00450-23-s0002.pdf Multiple sequence alignment of the known mcr genes with the four nucleic acid sequences in this study.

Legends msystems.00450-23-s0003.docx Legends for supplemental figures and tables.

Table S1 msystems.00450-23-s0004.xlsx The metadata and genomic information of *Cronobacter* carrying mcr genes in this study.

Table S2 msystems.00450-23-s0005.xlsx The genomic statistics of *Cronobacter* carrying mcr genes in this study.

Table S3 msystems.00450-23-s0006.xlsx The *Cronobacter* genomes from NCBI database.

Table S4 msystems.00450-23-s0007.xlsx The metadata of genome survey of *Cronobacter* genomes in this study.

ASM does not own the copyrights to Supplemental Material that may be linked to, or accessed through, an article. The authors have granted ASM a non-exclusive, world-wide license to publish the Supplemental Material files. Please contact the corresponding author directly for reuse.

## References

[1] Kift EV , Maartens G , Bamford C . 2014. Systematic review of the evidence for rational dosing of colistin. S Afr Med J 104:183–186. doi:10.7196/samj.7011 24897820

[2] Falagas ME , Kasiakou SK . 2005. Colistin: the revival of polymyxins for the management of multidrug-resistant gram-negative bacterial infections. Clin Infect Dis 40:1333–1341. doi:10.1086/429323 15825037

[3] Liu Y-Y , Wang Y , Walsh TR , Yi L-X , Zhang R , Spencer J , Doi Y , Tian G , Dong B , Huang X , Yu L-F , Gu D , Ren H , Chen X , Lv L , He D , Zhou H , Liang Z , Liu J-H , Shen J . 2016. Emergence of plasmid-mediated colistin resistance mechanism mcr-1 in animals and human beings in China: a microbiological and molecular biological study. Lancet Infect Dis 16:161–168. doi:10.1016/S1473-3099(15)00424-7 26603172

[4] Xavier BB , Lammens C , Ruhal R , Kumar-Singh S , Butaye P , Goossens H , Malhotra-Kumar S . 2016. Identification of a novel plasmid-mediated colistin-resistance gene, mcr-2, in Escherichia coli, Belgium, June 2016. Euro Surveill 21. doi:10.2807/1560-7917.ES.2016.21.27.30280 27416987

[5] Yin W , Li H , Shen Y , Liu Z , Wang S , Shen Z , Zhang R , Walsh TR , Shen J , Wang Y , Bush K . 2017. Novel plasmid-mediated colistin resistance gene mcr-3 in Escherichia coli. mBio 8. doi:10.1128/mBio.00543-17 PMC5487729 28655818

[6] Carattoli A , Villa L , Feudi C , Curcio L , Orsini S , Luppi A , Pezzotti G , Magistrali CF . 2017. Novel plasmid-mediated colistin resistance mcr-4 gene in Salmonella and Escherichia coli, Italy 2013, Spain and Belgium, 2015 to 2016. Euro Surveill 22:30589. doi:10.2807/1560-7917.ES.2017.22.31.30589 28797329 PMC5553062

[7] Borowiak M , Fischer J , Hammerl JA , Hendriksen RS , Szabo I , Malorny B . 2017. Identification of a novel transposon-associated phosphoethanolamine transferase gene, mcr-5, conferring colistin resistance in d-tartrate fermenting Salmonella enterica subsp. enterica serovar Paratyphi B. J Antimicrob Chemother 72:3317–3324. doi:10.1093/jac/dkx327 28962028

[8] AbuOun M , Stubberfield EJ , Duggett NA , Kirchner M , Dormer L , Nunez-Garcia J , Randall LP , Lemma F , Crook DW , Teale C , Smith RP , Anjum MF . 2017. mcr-1 and mcr-2 variant genes identified in Moraxella species isolated from pigs in Great Britain from 2014 to 2015. J Antimicrob Chemother 72:2745–2749. doi:10.1093/jac/dkx286 29091227 PMC5890717

[9] Yang Y-Q , Li Y-X , Lei C-W , Zhang A-Y , Wang H-N . 2018. Novel plasmid-mediated colistin resistance gene mcr-7.1 in Klebsiella pneumoniae. J Antimicrob Chemother 73:1791–1795. doi:10.1093/jac/dky111 29912417

[10] Wang X , Wang Y , Zhou Y , Li J , Yin W , Wang S , Zhang S , Shen J , Shen Z , Wang Y . 2018. Emergence of a novel mobile colistin resistance gene, mcr-8, in NDM-producing Klebsiella pneumoniae. Emerg Microbes Infect 7:122. doi:10.1038/s41426-018-0124-z 29970891 PMC6030107

[11] Carroll LM , Gaballa A , Guldimann C , Sullivan G , Henderson LO , Wiedmann M . 2019. Identification of novel mobilized colistin resistance gene mcr-9 in a multidrug-resistant, colistin-susceptible Salmonella enterica serotype typhimurium isolate. mBio 10:e00853-19. doi:10.1128/mBio.00853-19 31064835 PMC6509194

[12] Wang C , Feng Y , Liu L , Wei L , Kang M , Zong Z . 2020. Identification of novel mobile colistin resistance gene mcr-10. Emerg Microbes Infect 9:508–516. doi:10.1080/22221751.2020.1732231 32116151 PMC7067168

[13] Sun J , Zhang H , Liu Y-H , Feng Y . 2018. Towards understanding mcr-like colistin resistance. Trends Microbiol 26:794–808. doi:10.1016/j.tim.2018.02.006 29525421

[14] Ling Z , Yin W , Shen Z , Wang Y , Shen J , Walsh TR . 2020. Epidemiology of mobile colistin resistance genes mcr-1 to mcr-9. J Antimicrob Chemother 75:3087–3095. doi:10.1093/jac/dkaa205 32514524

[15] Nang SC , Li J , Velkov T . 2019. The rise and spread of mcr plasmid-mediated polymyxin resistance. Crit Rev Microbiol 45:131–161. doi:10.1080/1040841X.2018.1492902 31122100 PMC6625916

[16] Simmons BP , Gelfand MS , Haas M , Metts L , Ferguson J . 1989. Enterobacter sakazakii infections in neonates associated with intrinsic contamination of a powdered infant formula. Infect Control Hosp Epidemiol 10:398–401. doi:10.1086/646060 2794464

[17] Biering G , Karlsson S , Clark NC , Jónsdóttir KE , Lúdvígsson P , Steingrímsson O . 1989. Three cases of neonatal meningitis caused by Enterobacter sakazakii in powdered milk. J Clin Microbiol 27:2054–2056. doi:10.1128/jcm.27.9.2054-2056.1989 2778070 PMC267737

[18] Clark NC , Hill BC , O’Hara CM , Steingrimsson O , Cooksey RC . 1990. Epidemiologic typing of Enterobacter sakazakii in two neonatal nosocomial outbreaks. Diagn Microbiol Infect Dis 13:467–472. doi:10.1016/0732-8893(90)90078-a 2279379

[19] Friedemann M . 2009. Epidemiology of invasive neonatal Cronobacter (Enterobacter sakazakii) infections. Eur J Clin Microbiol Infect Dis 28:1297–1304. doi:10.1007/s10096-009-0779-4 19662446

[20] Liu B-T , Song F-J , Zou M , Hao Z-H , Shan H . 2017. Emergence of colistin resistance gene mcr-1 in Cronobacter sakazakii producing NDM-9 and in Escherichia coli from the same animal. Antimicrob Agents Chemother 61:e01444-16. doi:10.1128/AAC.01444-16 27855074 PMC5278688

[21] Yang J , Liu L , Feng Y , He D , Wang C , Zong Z . 2021. Potential mobilization of mcr-10 by an integrative mobile element via site-specific recombination in Cronobacter sakazakii. Antimicrob Agents Chemother 65:e01717-20. doi:10.1128/AAC.01717-20 33199393 PMC7849017

[22] Seo KH , Brackett RE . 2005. Rapid, specific detection of Enterobacter sakazakii in infant formula using a real-time PCR assay. J Food Prot 68:59–63. doi:10.4315/0362-028x-68.1.59 15690804

[23] Ewels P , Magnusson M , Lundin S , Käller M . 2016. MultiQC: summarize analysis results for multiple tools and samples in a single report. Bioinformatics 32:3047–3048. doi:10.1093/bioinformatics/btw354 27312411 PMC5039924

[24] Bolger AM , Lohse M , Usadel B . 2014. Trimmomatic: a flexible trimmer for Illumina sequence data. Bioinformatics 30:2114–2120. doi:10.1093/bioinformatics/btu170 24695404 PMC4103590

[25] Bankevich A , Nurk S , Antipov D , Gurevich AA , Dvorkin M , Kulikov AS , Lesin VM , Nikolenko SI , Pham S , Prjibelski AD , Pyshkin AV , Sirotkin AV , Vyahhi N , Tesler G , Alekseyev MA , Pevzner PA . 2012. SPAdes: a new genome assembly algorithm and its applications to single-cell sequencing. J Comput Biol 19:455–477. doi:10.1089/cmb.2012.0021 22506599 PMC3342519

[26] Seemann T . 2014. Prokka: rapid prokaryotic genome annotation. Bioinformatics 30:2068–2069. doi:10.1093/bioinformatics/btu153 24642063

[27] Wood DE , Salzberg SL . 2014. Kraken: ultrafast metagenomic sequence classification using exact alignments. Genome Biol 15:R46. doi:10.1186/gb-2014-15-3-r46 24580807 PMC4053813

[28] Schwengers O , Barth P , Falgenhauer L , Hain T , Chakraborty T , Goesmann A . 2020. Platon: identification and characterization of bacterial plasmid contigs in short-read draft assemblies exploiting protein sequence-based replicon distribution scores. Microb Genom 6:mgen000398. doi:10.1099/mgen.0.000398 32579097 PMC7660248

[29] Page AJ , Cummins CA , Hunt M , Wong VK , Reuter S , Holden MTG , Fookes M , Falush D , Keane JA , Parkhill J . 2015. Roary: rapid large-scale prokaryote pan genome analysis. Bioinformatics 31:3691–3693. doi:10.1093/bioinformatics/btv421 26198102 PMC4817141

[30] Price MN , Dehal PS , Arkin AP . 2009. FastTree: computing large minimum evolution trees with profiles instead of a distance matrix. Mol Biol Evol 26:1641–1650. doi:10.1093/molbev/msp077 19377059 PMC2693737

[31] Edgar RC . 2004. MUSCLE: multiple sequence alignment with high accuracy and high throughput. Nucleic Acids Res 32:1792–1797. doi:10.1093/nar/gkh340 15034147 PMC390337

[32] Letunic I , Bork P . 2019. Interactive Tree Of Life (iTOL) v4: recent updates and new developments. Nucleic Acids Res 47:W256–W259. doi:10.1093/nar/gkz239 30931475 PMC6602468

[33] Alcock BP , Raphenya AR , Lau TTY , Tsang KK , Bouchard M , Edalatmand A , Huynh W , Nguyen A-LV , Cheng AA , Liu S , Min SY , Miroshnichenko A , Tran H-K , Werfalli RE , Nasir JA , Oloni M , Speicher DJ , Florescu A , Singh B , Faltyn M , Hernandez-Koutoucheva A , Sharma AN , Bordeleau E , Pawlowski AC , Zubyk HL , Dooley D , Griffiths E , Maguire F , Winsor GL , Beiko RG , Brinkman FSL , Hsiao WWL , Domselaar GV , McArthur AG . 2020. CARD 2020: antibiotic resistome surveillance with the comprehensive antibiotic resistance database. Nucleic Acids Res 48:D517–D525. doi:10.1093/nar/gkz935 31665441 PMC7145624

[34] Siguier P , Perochon J , Lestrade L , Mahillon J , Chandler M . 2006. ISfinder: the reference centre for bacterial insertion sequences. Nucleic Acids Res 34:D32–D36. doi:10.1093/nar/gkj014 16381877 PMC1347377

[35] Sullivan MJ , Petty NK , Beatson SA . 2011. Easyfig: a genome comparison visualizer. Bioinformatics 27:1009–1010. doi:10.1093/bioinformatics/btr039 21278367 PMC3065679

[36] He S , Shi X . 2021. Microbial food safety in China: past, present, and future. Foodborne Pathog Dis 18:510–518. doi:10.1089/fpd.2021.0009 34242111

[37] Havelaar AH , Brul S , de Jong A , de Jonge R , Zwietering MH , Ter Kuile BH . 2010. Future challenges to microbial food safety. Int J Food Microbiol 139 Suppl 1:S79–S94. doi:10.1016/j.ijfoodmicro.2009.10.015 19913933

[38] Fusco V , Chieffi D , Fanelli F , Logrieco AF , Cho G-S , Kabisch J , Böhnlein C , Franz C . 2020. Microbial quality and safety of milk and milk products in the 21st century. Compr Rev Food Sci Food Saf 19:2013–2049. doi:10.1111/1541-4337.12568 33337106

[39] Jang H , Eshwar A , Lehner A , Gangiredla J , Patel IR , Beaubrun J-G , Chase HR , Negrete F , Finkelstein S , Weinstein LM , Ko K , Addy N , Ewing L , Woo J , Lee Y , Seo K , Jaradat Z , Srikumar S , Fanning S , Stephan R , Tall BD , Gopinath GR . 2022. Characterization of Cronobacter sakazakii strains originating from plant-origin foods using comparative genomic analyses and zebrafish infectivity studies. Microorganisms 10:1396. doi:10.3390/microorganisms10071396 35889115 PMC9319161

[40] Parra-Flores J , Holy O , Acuna S , Lepuschitz S , Pietzka A , Contreras-Fernandez A , Chavarria-Sepulveda P , Cruz-Cordova A , Xicohtencatl-Cortes J , Mancilla-Rojano J , Castillo A , Ruppitsch W , Forsythe S S . 2022. Genomic characterization of Cronobacter spp. and Salmonella spp. strains isolated from powdered Infant formula in Chile. Front Microbiol 13:884721. doi:10.3389/fmicb.2022.884721 35722296 PMC9201451

[41] Parra-Flores J , Holý O , Riffo F , Lepuschitz S , Ruppitsch W , Forsythe S . 2021. Draft genome sequences of seven Cronobacter sakazakii strains carrying the mcr 9.1 gene isolated in Chile. Microbiol Resour Announc 10:e0050621. doi:10.1128/MRA.00506-21 34264101 PMC8280878

[42] Akineden Ö , Heinrich V , Gross M , Usleber E . 2017. Reassessment of Cronobacter spp. originally isolated as Enterobacter sakazakii from infant food. Food Microbiol 65:44–50. doi:10.1016/j.fm.2017.01.021 28400018

[43] Phair K , Pereira SG , Kealey C , Fanning S , Brady DB . 2022. Insights into the mechanisms of Cronobacter sakazakii virulence. Microb Pathog 169:105643. doi:10.1016/j.micpath.2022.105643 35716925

[44] Xu T , Zhang C , Ji Y , Song J , Liu Y , Guo Y , Zhou K . 2021. Identiﬁcation of mcr-10 carried by self-transmissible plasmids and chromosome in Enterobacter roggenkampii strains isolated from hospital sewage water. Environ Pollut 268:115706. doi:10.1016/j.envpol.2020.115706 33069047

