# Supplementary figures and images for: Large-scale genomic survey and characterization of mcr genes carried by foodborne Cronobacter isolates
Source: mSystems. 2023 Sep 11;8(5):e00450-23. doi: 10.1128/msystems.00450-23 (PMC10654070; doi:10.1128/msystems.00450-23)

Number of Strains with ARGs

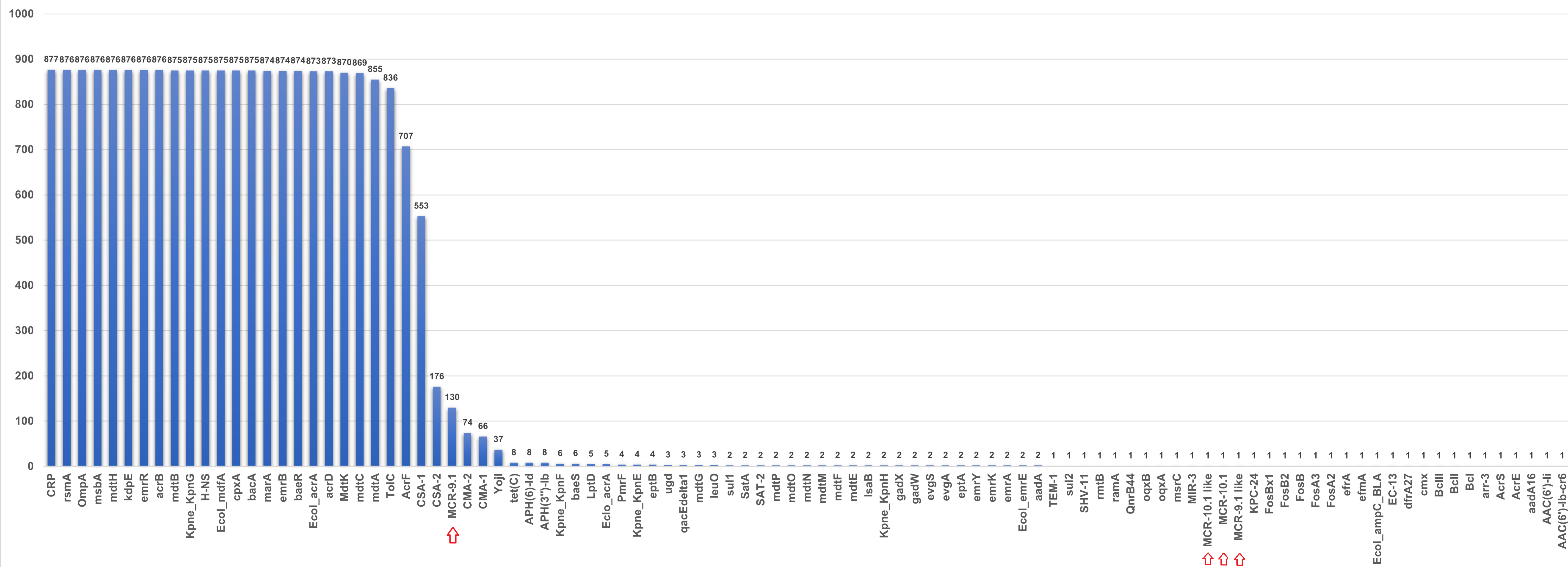

Supplement: Figure S1 — Antibiotic resistance profile of Cronobacter isolates. [file msystems.00450-23-s0001.pdf]

A

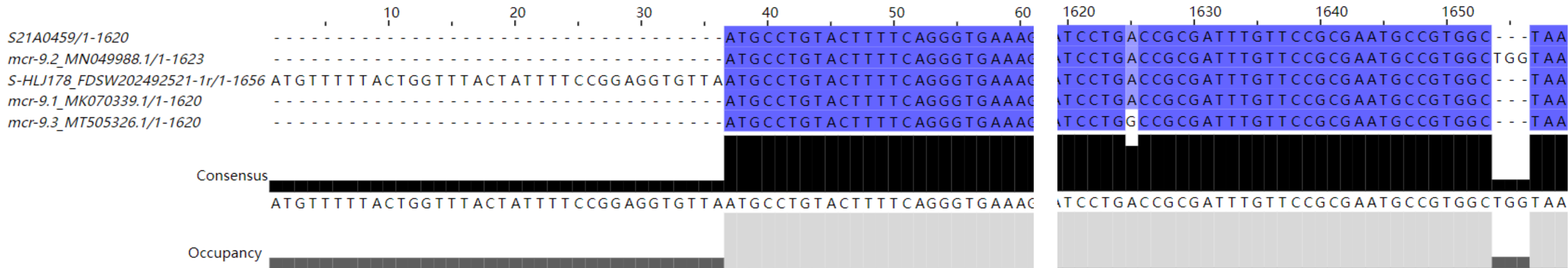

B

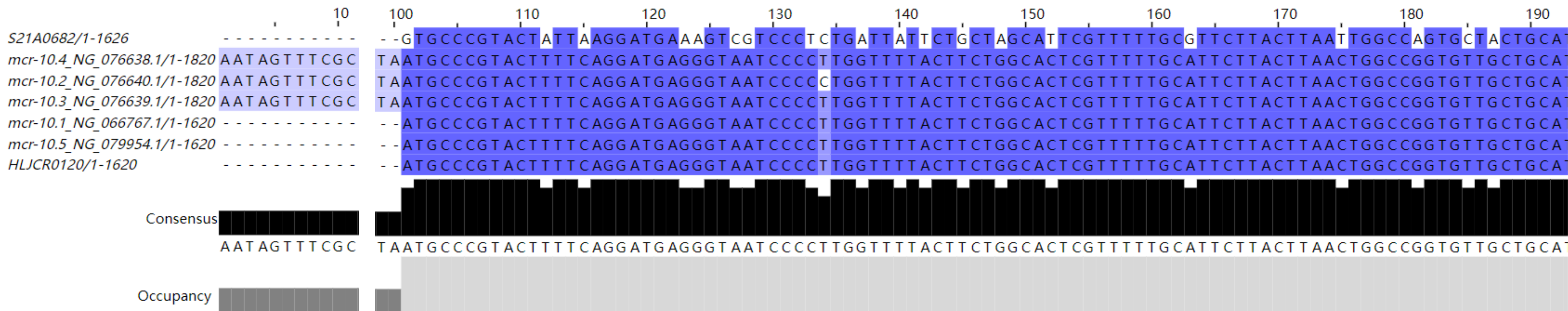

Supplement: Figure S2 — Multiple sequence alignment of the known mcr genes with the four nucleic acid sequences in this study. [file msystems.00450-23-s0002.pdf]
