# Supplementary material for: Large-scale genomic survey and characterization of mcr genes carried by foodborne Cronobacter isolates
Source: mSystems. 2023 Sep 11;8(5):e00450-23. doi: 10.1128/msystems.00450-23 (PMC10654070; doi:10.1128/msystems.00450-23)
Supplement: Legends — for supplemental figures and tables. [file msystems.00450-23-s0003.docx]

**Supplementary materials**

Figure S1 Antibiotic resistance profile of *Cronobacter* isolates.

Figure S2 Multiple sequence alignment of the known mcr genes with the four nucleic acid sequences in this study.

Table S1 The metadata and genomic information of *Cronobacter* carring mcr genes in this study.

Table S2 The genomic statistics of *Cronobacter* carring mcr genes in this study.

Table S3 The *Cronobacter* genomes from NCBI database.

Table S4 The metadata of genome survey of *Cronobacter* genomes in this study.
